# Stearoyl-CoA Desaturase-1 Protects Cells against Lipotoxicity-Mediated Apoptosis in Proximal Tubular Cells

**DOI:** 10.3390/ijms17111868

**Published:** 2016-11-09

**Authors:** Tamaki Iwai, Shinji Kume, Masami Chin-Kanasaki, Shogo Kuwagata, Hisazumi Araki, Naoko Takeda, Takeshi Sugaya, Takashi Uzu, Hiroshi Maegawa, Shin-ichi Araki

**Affiliations:** 1 Department of Medicine, Shiga University of Medical Science, Tsukinowa-cho, Seta, Otsu, Shiga 520-2192, Japan; tama@belle.shiga-med.ac.jp (T.I.); msm@belle.shiga-med.ac.jp (M.C.-K.); skuwaga@belle.shiga-med.ac.jp (S.K.); haraki@belle.shiga-med.ac.jp (H.A.); takedan@belle.shiga-med.ac.jp (N.T.); takuzu@belle.shiga-med.ac.jp (T.U.); maegawa@belle.shiga-med.ac.jp (H.M.); 2 Department of Internal Medicine, St. Marianna University School of Medicine, Kawasaki, Kanagawa 216-8511, Japan; takeshi-sugaya@marianna-u.ac.jp

**Keywords:** lipotoxicity, desaturation, free fatty acid, proximal tubular epithelial cells, lipid droplet

## Abstract

Saturated fatty acid (SFA)-related lipotoxicity is a pathogenesis of diabetes-related renal proximal tubular epithelial cell (PTEC) damage, closely associated with a progressive decline in renal function. This study was designed to identify a free fatty acid (FFA) metabolism-related enzyme that can protect PTECs from SFA-related lipotoxicity. Among several enzymes involved in FFA metabolism, we identified stearoyl-CoA desaturase-1 (SCD1), whose expression level significantly decreased in the kidneys of high-fat diet (HFD)-induced diabetic mice, compared with non-diabetic mice. SCD1 is an enzyme that desaturates SFAs, converting them to monounsaturated fatty acids (MUFAs), leading to the formation of neutral lipid droplets. In culture, retrovirus-mediated overexpression of *SCD1* or MUFA treatment significantly ameliorated SFA-induced apoptosis in PTECs by enhancing intracellular lipid droplet formation. In contrast, siRNA against *SCD1* exacerbated the apoptosis. Both overexpression of *SCD1* and MUFA treatment reduced SFA-induced apoptosis via reducing endoplasmic reticulum stress in cultured PTECs. Thus, HFD-induced decrease in renal SCD1 expression may play a pathogenic role in lipotoxicity-induced renal injury, and enhancing SCD1-mediated desaturation of SFA and subsequent formation of neutral lipid droplets may become a promising therapeutic target to reduce SFA-induced lipotoxicity. The present study provides a novel insight into lipotoxicity in the pathogenesis of diabetic nephropathy.

## 1. Introduction

In diabetic nephropathy, proteinuria is a leading cause of tubulointerstitial lesions and subsequent renal dysfunction, as well as a marker of glomerular lesions [1,2]. Reducing proteinuria is therefore considered a principal therapeutic target. Clinical evidence has shown that intensive treatment with strict glycemic control and blood pressure control using a renin angiotensin system blockade can successfully reduce or abrogate proteinuria [3,4,5,6]. Unfortunately, however, some patients still develop refractory proteinuria, resulting in end stage renal disease. Thus, identifying a new therapy for these patients is urgently needed.

Given that, regardless of underlying diseases, the renal prognosis is correlated with the severity of the tubulointerstitial lesions, protecting proximal tubular epithelial cells (PTECs) from proteinuria-induced cytotoxicity should be the next therapeutic target to improve renal prognosis in refractory proteinuria. Clinical studies have shown that renal prognosis is extremely poor in diabetic nephropathy [7]. Furthermore, we have previously reported that the PTECs in diabetic mice were more susceptible to proteinuria-related toxicity [8]. These observations suggest that a certain diabetes-related systemic and/or renal change exacerbate proteinuria-induced PTEC damage, leading to poor renal prognosis, and that identifying the details in vulnerable diabetes-related PTECs may lead to an additional therapy that will improve the renal prognosis of refractory diabetic nephropathy.

Serum free fatty acids (FFAs) are composed of saturated fatty acids (SFAs), monounsaturated fatty acids (MUFAs) and polyunsaturated fatty acids (PUFAs) [9]. In obese or type 2 diabetes patients, the serum FFA profile includes higher SFAs, which is more cytotoxic than unsaturated FAs [10,11,12]. Thus, the systemic change in FFA metabolism has recently been highlighted in tissue damage in various organs of type 2 diabetes patients [13,14,15]. As serum FFAs are bound to albumin in the blood, FFA-bound albumin filtered through the injured glomeruli is reabsorbed by PTECs, promoting severe tubulointerstitial injury [16,17]. Indeed, the urinary FFA level is higher in diabetic nephropathy [18]. Thus, renal lipotoxicity due to urinary SFA-bound albumin is considered an aggressive factor that develops diabetes-related severe PTEC damage.

Another type of renal lipotoxicity has also been reported to be associated with the pathogenesis of diabetic nephropathy. Growing evidence demonstrates that intra-renal lipid metabolism is altered in diabetic patients and animals, including enhanced lipogenesis and suppressed lipolysis, and that normalization of the alteration is essential for reducing FFA-mediated cytotoxicity [19,20,21,22,23]. These observations led us to hypothesize that the local alteration in FFA metabolism in diabetic PTECs impairs an adaptive response against the excess inflow of urinary SFA-bound albumin. If this hypothesis is correct, revealing a unique renal lipid metabolism alteration associated with the exacerbation of SFA-mediated PTEC damage should provide a novel strategy for protecting PTECs from persistent proteinuria in diabetic nephropathy.

To this end, this study was designed to reveal an FFA metabolism-related enzyme that is altered in diabetic PTECs and can inhibit SFA-mediated tubular cell damage. The present study demonstrated that downregulation of stearoyl-Coenzyme A desaturase-1 (SCD1) in diabetic PTECs is associated with cell vulnerability to the cytotoxicity of SFA-bound albumin, suggesting that activation of the enzyme may become a novel therapy for protecting PTECs from refractory proteinuria in diabetic nephropathy.

## 2. Results

### 2.1. Renal Lipid Metabolism Is Altered in the Kidneys of High-Fat Diet (HFD)—Induced Obese Type 2 Diabetic Mice

**Figure 1:**
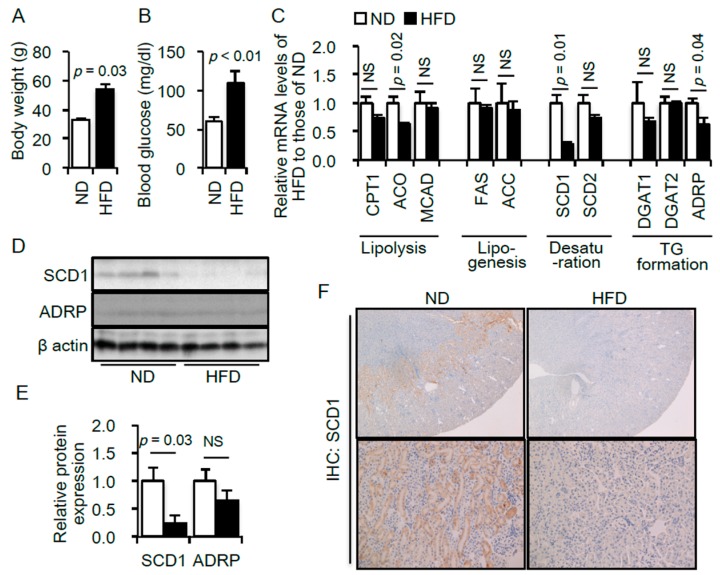
Body weight (**A**) and blood glucose levels (**B**) of mice fed with either normal diet (ND, *n* = 5) or high-fat diet (HFD, *n* = 5); (**C**) Expression levels of mRNAs related to lipid metabolism in the kidneys of mice fed an ND or a HFD. Data are expressed as a relative ratio to the ND group; (**D**) Representative immunoblot of SCD1 and ADRP. β-actin was used as a loading control; (**E**) Quantitative results of the immunoblots. Data are expressed as a relative ratio to the ND group. *p* < 0.05 indicates statistical significance; (**F**) Immunohistological analysis of SCD1 in kidney sections of mice fed an ND or a HFD. Magnification: ×40 (**upper**), ×200 (**lower**). Data are shown as mean ± SD. *p* < 0.05 indicates statistical significance. NS indicates no significance. CPT1, carnitine palmitoyltransferase 1; ACO, acetyl coenzyme A oxidase; MCAD, medium-chain acyl-CoA dehydrogenase; FAS, fatty acid synthase; ACC, acetyl-CoA carboxylase; SCD, stearoyl-CoA desaturase; DGAT, diacylglycerol transferase; ADRP, adipose differentiation-related protein.

Because FFA metabolism includes several pathways, including lipolysis, lipogenesis, desaturation and triglyceride (TG) formation, we first examined the change in the mRNA expression levels of various genes related to each pathway in the kidneys of diabetic mice. The obese type 2 diabetes mouse model was induced by HFD feeding for 8 weeks. Significant increases in body weight gain and blood glucose were confirmed in the model (Figure 1A,B). We screened for genes whose mRNA expression levels were altered in the kidney samples of HFD-fed mice. Among the genes associated with FFA metabolism, the mRNA expression levels of *ACO*, *SCD1* and *ADRP* were significantly decreased in the kidneys of HFD-fed obese type 2 diabetic mice (Figure 1C).

Of these three genes, the renoprotective role of lipolytic enzymes including ACO has been reported [24]. We thus examined the protein expression levels of SCD1 and ADRP in the mouse kidney. The protein expression level of SCD1, but not ADRP, was significantly decreased in the kidneys of HFD-fed mice (Figure 1D,E). Furthermore, a marked decrease in SCD1 expression in the kidneys of HFD-fed mice was confirmed by an immunohistochemical experiment (Figure 1F). Abundant expression of SCD1 in the renal corticomedullary and cortex regions was found in normal kidneys, which dramatically decreased in the kidney of HFD-fed mice (Figure 1F).

### 2.2. SCD1 Overexpression Ameliorates Saturated FA-Induced Apoptosis in Cultured Proximal Tubular Cells

**Figure 2:**
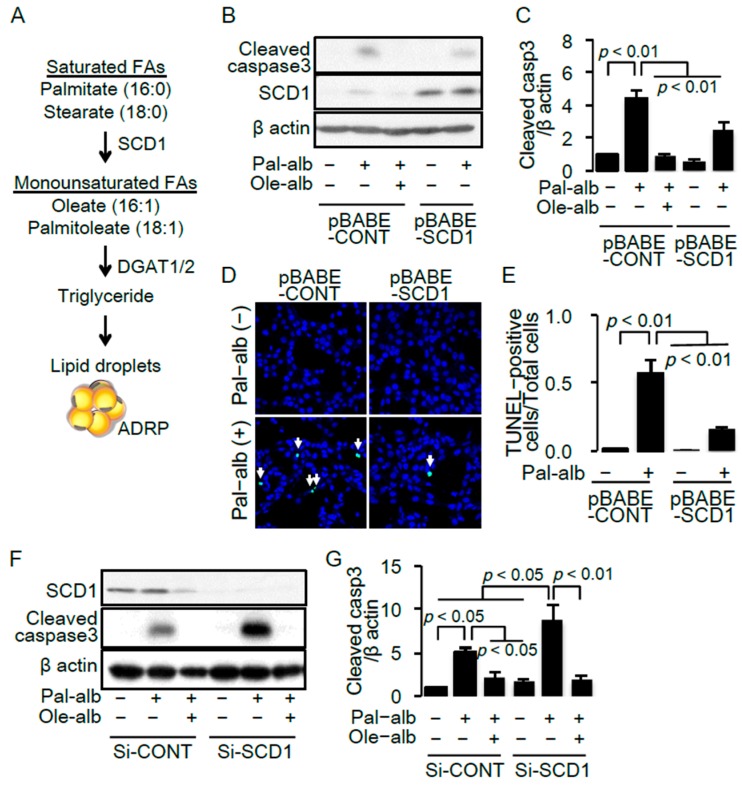
(**A**) Schema representing the formation of neutral lipid droplets from saturated free fatty acids (FAs) such as palmitate and stearate; (**B**) Representative immunoblot of cleaved caspase 3 and SCD1 in cultured proximal tubular cells infected with the control retrovirus (pBABE-CONT) or the retrovirus for *SCD1* overexpression (pBABE-SCD1), and treated with/without palmitate or oleate. β-actin was used as a loading control; (**C**) Quantitative result of cleaved caspase 3 (*n* = 5); (**D**) Representative pictures of TUNEL staining under the indicated conditions. White arrows indicate TUNEL-positive apoptotic cells; (**E**) Quantitative results of TUNEL staining; (**F**) Representative immunoblot of cleaved caspase 3 and SCD1 in cultured proximal tubular cells transfected with the control siRNA or siRNA against *SCD1*, and treated with/without palmitate or oleate; (**G**) Quantitative result of cleaved caspase 3 (*n* = 7). SCD, stearoyl-CoA desaturase. Data are shown as mean ± SD. *p* < 0.05 indicates statistical significance.

SCD1 synthesizes MUFAs from SFAs, which is necessary for the biosynthesis of triglycerides [25] (Figure 2A). To examine a significance of the decrease in SCD1 expression in the kidney of HFD mice, we generated a proximal tubular cell line overexpressing SCD1 by a retrovirus-mediated gene transfer system. Stimulation with saturated FA palmitate-bound albumin significantly induced apoptosis determined by cleavage of caspase 3 in the cultured proximal tubular cells, which was significantly ameliorated by co-treatment with either the monounsaturated FA, oleate, or by overexpression of SCD1 (Figure 2B,C). The anti-apoptotic effect of SCD1 overexpression was confirmed by terminal deoxynucleotidyl transferase dUTP nick end labeling (TUNEL) staining (Figure 2D,E).

Furthermore, siRNA against the *SCD1* gene significantly enhanced the palmitate-induced cleavage of caspase 3, which was completely abolished by co-treatment with oleate (Figure 2F,G). These results indicated that an imbalance between saturated and monounsaturated FAs is associated with cell death and suggests that decreased SCD1 expression in HFD mice played a pathogenic role in renal injury associated with obesity.

### 2.3. SCD1 Overexpression Decreases Saturated FA-Induced Endoplasmic Reticulum Stress in Cultured Proximal Tubular Cells

**Figure 3:**
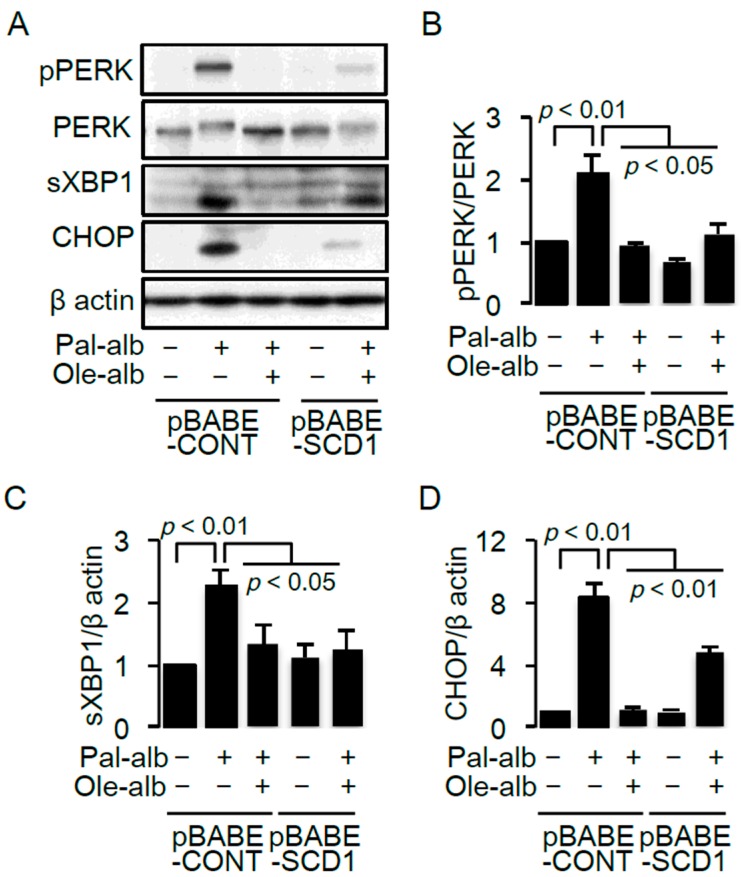
(**A**) Representative immunoblot of pPERK, PERK, sXBP1 and CHOP in cultured proximal tubular cells infected with the control retrovirus (pBABE-CONT) or the retrovirus for SCD1 overexpression (pBABE-SCD1), and treated with/without palmitate or oleate. β-actin was used as a loading control. Quantitative results of the pPERK/PERK ratio (**B**), sXBP1 (**C**) and CHOP (**D**) (*n* = 4). Data are shown as mean ± SD. *p* < 0.05 indicates statistical significance. pPERK, PKR-like ER kinase (PERK) phosphorylated at Thr980; sXBP1, spliced X-box binding protein 1; CHOP, C/EBP homologous protein; SCD, stearoyl-CoA desaturase.

Endoplasmic reticulum (ER) stress is strongly associated with saturated FA-induced apoptosis in a variety of cells [26,27,28]. Enhanced ER stress, determined by protein kinase RNA-like endoplasmic reticulum kinase (PERK) phosphorylation and XBP1 splicing, was confirmed in cultured proximal tubular cells exposed to palmitate, accompanied by overexpression of CCAAT-enhancer-binding protein homologous protein (CHOP), a transcriptional factor responsible for ER stress-induced apoptosis (Figure 3A−D). The palmitate-bound albumin-induced increase in ER stress was significantly inhibited by either oleate treatment or a retroviral-mediated overexpression of SCD1 (Figure 3A−D).

### 2.4. SCD1 Overexpression Enhances Neutral Lipid Droplet Formation in Cultured Proximal Tubular Cells Exposed to Saturated FAs

**Figure 4:**
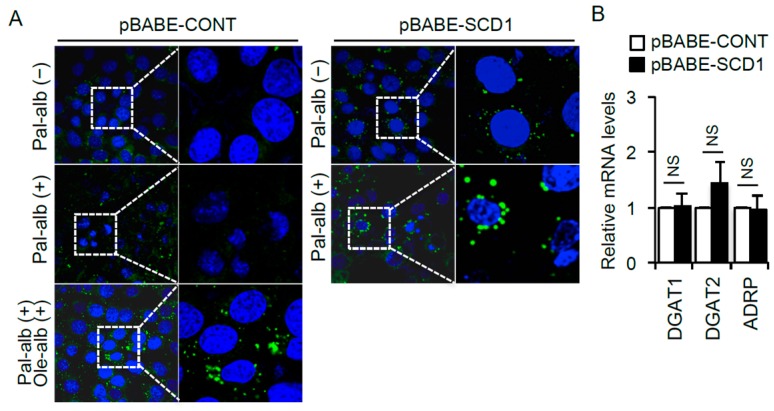
(**A**) Representative BODIPY staining for visualizing intracellular neutral lipid accumulation in cultured proximal tubular cells infected with the control retrovirus (pBABE-CONT) or the retrovirus for *SCD1* overexpression (pBABE-SCD1), and treated with/without palmitate or oleate; (**B**) mRNA expression levels of *DGAT1*, *DGAT2* and *ADRP* (*n* = 6). Data are shown as mean ± SD. NS indicates no significance. DGAT, diacylglycerol transferase; ADRP, adipose differentiation-related protein.

SCD1 expression is critical for desaturation of FAs and subsequent triglyceride formation in cells (Figure 2A). We next examined the role of lipid droplet formation in palmitate-bound albumin-induced apoptosis in cultured proximal tubular cells. Stimulation with palmitate alone did not form lipid droplets in the cells; however, co-stimulation with oleate markedly increased lipid droplet formation (Figure 4A). Similar to the oleate effect, SCD1 overexpression increased intracellular lipid droplet formation (Figure 4A), without affecting the gene expression levels associated with lipid droplet formation (Figure 4B).

**Figure 5:**
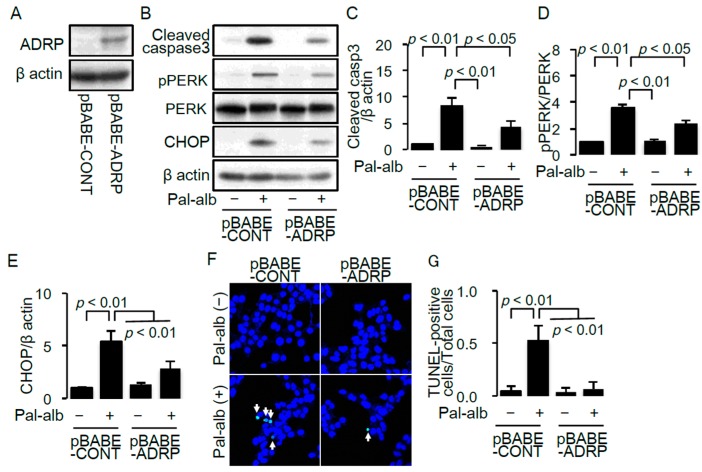
(**A**) Representative immunoblot of ADRP in cultured proximal tubular cells infected with the control retrovirus (pBABE-CONT) or the retrovirus for *ADRP* overexpression (pBABE-ADRP); (**B**) Representative immunoblot of cleaved caspase 3, pPERK, PERK and CHOP in pBABE-CONT or pBABE-ADRP transfected cells, treated with/without palmitate. β-actin was used as a loading control; Quantitative results of cleaved caspase 3 (**C**), pPERK/PERK ratio (**D**) and CHOP (**E**) (*n* = 4); (**F**) Representative pictures of TUNEL staining for detecting apoptotic cells in pBABE-CONT or pBABE-ADRP transfected cells, treated with/without palmitate. White arrows indicate TUNEL-positive apoptotic cells; (**G**) Quantitative results of TUNEL staining. Data are shown as mean ± SD. *p* < 0.05 indicates statistical significance. ADRP, adipose differentiation-related protein; pPERK, PKR-like ER kinase (PERK) phosphorylated at Thr980; CHOP, C/EBP homologous protein; SCD, stearoyl-CoA desaturase.

To confirm the anti-apoptotic effect of lipid droplet formation in cells treated with saturated FA-bound albumin, we generated a cell line overexpressing ADRP [29]. ADRP overexpression was confirmed by western blot analysis (Figure 5A). Overexpression of ADRP significantly decreased caspase 3 cleavage (Figure 5B,C) and the number of TUNEL-positive apoptotic cells (Figure 5C,F). Furthermore, overexpression of ADRP ameliorated the palmitate-bound albumin-induced ER stress characterized by increased PERK phosphorylation and CHOP expression (Figure 5B,D,E). These results suggest that enhancing lipid droplet formation has a cytoprotective role against saturated FA-mediated cell toxicity.

## 3. Discussion

In this study, we identified SCD1, whose expression level decreased in diabetic kidneys, as a candidate FFA metabolism enzyme that can ameliorate the SFA-mediated PTEC damage. Furthermore, SCD1-mediated enhancement of neutral lipid formation was associated with protection of PTECs from SFA-mediated PTEC damage.

Several reports have shown that altered intra-renal FFA metabolism is associated with the pathogenesis of diabetic nephropathy [19,20,21,22,23,24]. Among these, decreased levels of lipolysis enzymes have been most extensively focused on as a therapeutic target for diabetic nephropathy [23]. In addition to intra-renal lipolysis, this study demonstrated, for the first time, that intra-renal desaturation of SFAs by SCD1 is also a therapeutic target for the prevention of PTEC damage in diabetic nephropathy. Furthermore, a previous report has shown that SCD1 activation has a cell-protective role in glomerular epithelial cells [30]. Given that SFA-mediated cellular toxicity contributes to both tubulointerstitial lesions and podocyte injury, stimulating SCD1 activity may become a novel therapeutic target for improving the renal prognosis by both reducing proteinuria and ameliorating PTEC damage in the refractory diabetic nephropathy.

Although the idea that unsaturated FAs show a renoprotective role is consistent with previous reports including ours [13,31], the present study, for the first time, showed that local impairment of fatty acid desaturation is associated with the pathogenesis of PTEC damage in diabetic nephropathy, and that the SCD1-mediated endogenous MUFA synthesis and subsequent neutral lipid production showed a cell-protective action against SFA-mediated PTEC damage via reducing ER stress. The effectiveness of omega-3 PUFA treatment or a Mediterranean diet, which is rich in MUFAs, has been suggested in the clinical setting. However, the effectiveness of these interventions is still under debate, because the clinical studies did not show positive results in the unsaturated FAs treatment group. In this study, renal SCD1 expression decreased in all diabetic mice. However, in the clinical setting, SCD1 expression may not be decreased in all diabetic patients, which may influence the discrepant results in the unsaturated FAs intervention studies. If SFA-mediated lipotoxicity is associated with severe tubular damage only in patients showing low levels of renal SCD1 expression, a screening strategy for finding the high-risk patients may help improve the unsaturated FA treatment-based therapy.

**Figure 6:**
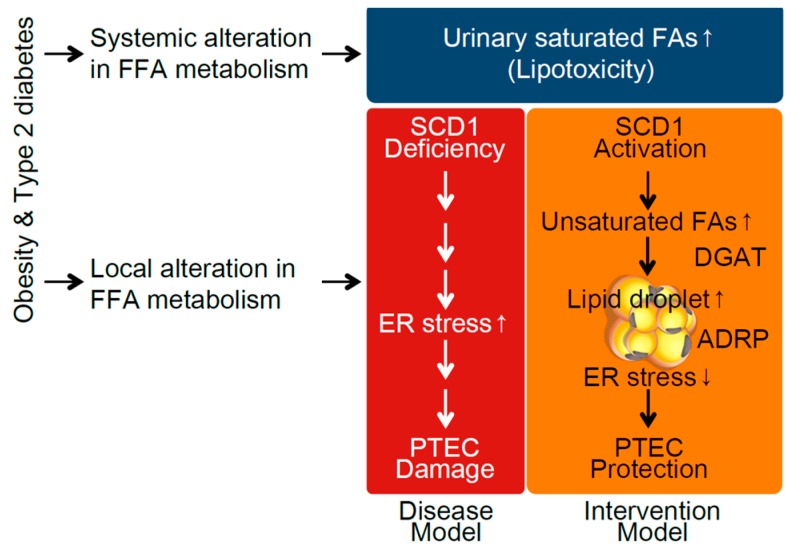
Schema showing the renoprotective mechanism of SCD1 against saturated fatty acid (SFA)-bound albumin-induced proximal tubular epithelial cell (PTEC) damage in type 2 diabetic nephropathy. Systemic alteration in FFA metabolism increases urinary SFA-related lipotoxicity. Furthermore, diabetic conditions alter intrarenal FA metabolism, characterized by SCD1 deficiency, enhancing ER stress-related PTEC damage. SCD1 activation may ameliorate SFA-induced PTEC damage via enhancing desaturation and subsequent lipid droplet formation.

Two types of renal lipotoxicity have been reported to be associated with the pathogenesis of diabetic nephropathy so far. One is urinary FFA-bound albumin-induced PTEC damage [16], which is mediated by systemic alteration in FFA metabolism. The other one is the alteration of intra-renal FFA metabolism [23]. The relationship between each of the two and the pathogenesis of diabetic complications has been independently mentioned so far. However, based on our results, there is a strong possibility that systemic and local tissue alterations in FFA metabolism synergistically contribute to the development of lipotoxicity-mediated cell damage in diabetes (Figure 6). This idea provides a novel research concept in diabetic complications. Hence, the interaction between systemic and local tissue changes in FFA metabolism should be more precisely investigated to establish a novel therapy for reducing lipotoxicity. Growing evidence suggests that SCD1 activation plays a cell-protective role in various metabolic tissues as well as kidney cells [32,33,34], and thus an SCD1 activator is expected to become a novel therapy for preventing disease progression associated with diabetes. However, SCD1 inhibition has also been suggested to be a new therapeutic target for preventing type 2 diabetes itself, because SCD1 knockout mice showed resistance to HFD-induced obesity and insulin resistance [35]. Thus, whether SCD1 should be systemically inhibited or activated in diabetic patients is still under debate, although SCD1 activation is likely to be a good candidate, at least in kidney cells under diabetic conditions. Furthermore, we demonstrated the effectiveness of SCD1 activation only in an in vitro cell culture study. To draw a final conclusion, further studies using kidney-specific SCD1 transgenic or knockout mice are required.

In conclusion, the alteration in renal FFA metabolism characterized by decreased SCD1 expression is associated with SFA-bound albumin-mediated lipotoxicity and apoptosis in diabetic PTECs. The results herein provide a novel insight into the lipotoxicity theory in diabetic nephropathy.

## 4. Experimental Procedures

### 4.1. Materials

Bovine serum albumin (BSA; fatty acid free, fraction V) was obtained from Nacalai Tesque (Kyoto, Japan). BSA did not contain a high level of endotoxin (<3.0 ng/mL), as confirmed by the Endospacy method (FALCO, Kyoto, Japan). Rabbit monoclonal antibody against cleaved caspase 3, rabbit monoclonal antibody against phosphorylated PERK, rabbit monoclonal antibody against PERK, mouse monoclonal antibody against CHOP, rabbit monoclonal antibody against SCD1 and rabbit monoclonal antibody against ADRP were from Cell Signaling Technology (Danvers, MA, USA), and rabbit polyclonal antibody against XBP1 was from Santa Cruz Biotechnology (Santa Cruz, CA, USA).

### 4.2. Animal Study

All procedures were performed in accordance with the guidelines of the Research Center for Animal Life Science of Shiga University of Medical Science, and followed the “3R”, “Replacement of animals by alternatives wherever possible”, “Reduction in number of animals used”, and “Refinement of experimental conditions and procedures to minimize the harm to animals” and the guidelines provided by the Animal Research: Reporting In Vivo Experiments (ARRIVE) guideline. The animal study was approved by the Research Center for Animal Life Science of Shiga University of Medical Science (No. 2013-4-5).

Seven-week-old male C57BL/6 mice were purchased from CLEA Japan (Tokyo, Japan), housed in cages and maintained on a 12 h light/dark cycle. The low-fat diet (ND: 10% of total calories from fat) and HFD (60% of total calories from fat) were purchased from Research Diets (New Brunswick, NJ, USA). After a week of acclimatization, mice were divided into a normal diet (ND) group (*n* = 5) and a HFD group (*n* = 5), and followed for 8 weeks. Blood and kidney samples were collected after 8 weeks of dietary intervention and stored at −80 °C until assayed. Fasting blood glucose concentrations were measured using a Glutest sensor (Sanwa Kagaku, Nagoya, Japan).

### 4.3. Immunohistochemistry

Fixed kidneys embedded in paraffin were sectioned (3-μm thickness), and immunohistochemical staining was performed with SCD1-specific rabbit polyclonal antibody (Cell Signaling Technology), as previously described [24].

### 4.4. Cell Culture

Murine proximal tubular (mProx24) cells were cultured as described previously [13]. In brief, cells ware cultured in Dulbecco’s modified Eagle medium (DMEM) containing 10% heat-inactivated fetal calf serum (FCS), 100 U/mL penicillin, and 100 µg/mL streptomycin. Cultured cells from passages 4–30 were used for the experiments. Subconfluent cells were turned quiescent by incubation with 10% FCS-DMEM for 12 h. Lipid-containing media were prepared by conjugating FFA with FFA-free BSA. Briefly, sodium palmitate (Sigma, St. Louis, MO, USA) and oleic acid (Sigma) were dissolved in 50% ethanol, mixed vigorously with FFA-free BSA in phosphate-buffered saline (PBS) at a molar ratio of 3:1, filter-sterilized, and added to culture media at a final FA concentration of 150 μmol/L (palmitate and oleate). The cells were incubated with the concentrations of FFA-bound albumin in the experimental medium (FBS-free DMEM) for 9 h in the indicated experiments.

### 4.5. Bodipy Stain

Lipid droplets in fixed cells were visualized with BODIPY 493/503 (Invitrogen, Carlsbad, CA, USA).

### 4.6. Retroviral-Mediated Gene Transfer

To generate a retroviral vector for gene overexpression, mouse *SCD1* cDNA and mouse *ADRP* cDNA were subcloned into pBABE vectors. Retroviral infection was performed as described previously [36]. HEK293T cells were transfected with pBABE-control, pBABE-SCD1 or pBABE-ADRP using lipofectamine reagent. At 48 h after transfection, the media containing retroviruses were collected, centrifuged and transferred to murine mesangial cells treated with polybrene (1 μg/mL). Infected cells were selected by treatment with puromycin (2.5 μg/mL) for several days.

### 4.7. siRNA Transfection

Cultured PTECs were seeded in 6-well plates and incubated for 24 h. Cells were transfected with 100 nM siRNA against *SCD1* (SMARTpool reagent; Dharmacon, Chicago, IL, USA) or control siRNA (nontargeting siRNA; Dharmacon) using DharmaFECT 4 transfection reagent (Dharmacon), and incubated for 24 h in 0.2% FCS-containing DMEM. Following transfection, the cells were starved for 24 h, treated with 150 μM palmitate-conjugated albumin and analyzed as indicated. The siRNA sequences are shown in Table 1.

### 4.8. TUNEL Assay

TUNEL staining was performed with an in situ apoptosis detection kit (Takara-Bio Inc., Otsu, Japan). TUNEL-positive and -negative cells were counted in four randomly selected areas of a culture slide. The results are expressed as a ratio of TUNEL-positive cells to total cell number in each field.

### 4.9. Measurements of mRNA Levels

mRNA levels were assessed by real-time polymerase chain reaction (PCR) [24]. Total RNA was isolated by TRIzol reagent (Invitrogen Life Technologies). cDNA was synthesized by reverse transcription PCR using reverse transcription reagents (Takara). Power SYBR Green Supermix (Bio-Rad Laboratories, Hercules, CA, USA) was used for real-time PCR (ABI Prism TM 7500 Sequence Detection System; PerkinElmer Applied Biosystems, Foster City, CA, USA). Analytical data were normalized against the *β-actin* mRNA expression level as an internal control. The sequences of the sense and antisense primers used for amplification are shown in the Table 2.

### 4.10. Immunoblot Analysis

Immunoblot analysis was performed as described previously [24]. Briefly, the cultured PTEC and kidney homogenates were lysed in SDS sample buffer (62.5 mM Tris-HCl, pH 6.8, 2% SDS, 10% glycerol, 50 mM dithiothreitol and 0.01% bromphenol blue). Forty micrograms of protein were electrophoresed on 12% SDS-polyacrylamide gels and electrotransferred onto polyvinylidene difluoride membranes (Immobilon, Millipore, Bedford, MA, USA). After the membranes were blocked, they were incubated with the indicated antibodies, washed and incubated with horseradish peroxidase-coupled secondary antibodies (Amersham, Buckinghamshire, UK). After washing, the blots were visualized by an enhanced chemiluminescence detection system (PerkinElmer, Boston, MA, USA).

### 4.11. Statistical Analysis

Results are expressed as mean±s.e.d. Analysis of variance with subsequent Tukey’s test was used to determine the significance of differences in multiple comparisons. A *p*-value <0.05 was considered statistically significant.

## Figures and Tables

**Table 1:** siRNA sequences used in this study.

si-RNA	Sequences
*NTC*	5’-ugguuuacaugucgacuaa-3’
*SCD1*	5’-ggaaguaauucgaguguau-3’
	5’-ccacugaauugcuauguua-3’
	5’-uccaagagaucuccaguuc-3’
	5’-ccgcgcaucucuauggaua-3’

**Table 2:** Primer sets used in the real-time PCR study.

Genes	Forward	Reverse
*β-actin*	5’-cgtgcgtgacatcaaagagaa-3’	5’-tggatgccacaggattccat-3’
*CPT-1*	5’-accactggccgaatgtcaag-3’	5’-agcgagtagcgcatggtcat-3’
*ACO*	5’-ggccaactatggtggacatca-3’	5’-accaatctggctgctgcacgaa-3’
*MCAD*	5’-taatcggtgaaggagcaggttt-3’	5’-ggcatacttcgtggcttcgt-3’
*FAS*	5’-tgtcctgcctctggtgctt-3’	5’-aatgggcctccttgatataatcct-3’
*ACC*	5’-cccagacagaataaagctactttgg-3’	5’-tccttttgtgcaactaggaacgt-3’
*SCD1*	5’-tgttgtccctatagcccaatccag-3’	5’-agctcagagcgcgtgttcaa-3’
*SCD2*	5’-agtgttgctcgtgagcctgtg-3’	5’-cctgcagatccatgtccagcta-3’
*DGAT1*	5’-ggtgcgagacgcggctgtga-3’	5’-agtagccgtcgcccacgctg-3’
*DGAT2*	5’-atgggtccagaagaagttccag-3’	5’-ggtgatgggcttggagtaggv-3’
*ADRP*	5’-tggcagcagcagtagtgga-3’	5’-acataagcggaggacacaagg-3’
